# Global Longitudinal Strain Predicts Outcomes in Patients with Reduced Left Ventricular Function Undergoing Transcatheter Edge-to-Edge Mitral Repair

**DOI:** 10.3390/jcm12124116

**Published:** 2023-06-18

**Authors:** Estefania Fernandez-Peregrina, Luis Asmarats, Rodrigo Estevez-Loureiro, Isaac Pascual, Diana Bastidas, Tomas Benito-González, Berenice Caneiro-Queija, Pablo Avanzas, Jose Alberto De Agustin, Felipe Fernández-Vazquez, Manuel Barreiro-Pérez, Victor Leon, Luis Nombela-Franco, Carmen Garrote, Chi-Hion Li, José Antonio Baz, Antonio Adeba, Jordi Sans-Roselló, Javier Gualis, Dabit Arzamendi

**Affiliations:** 1 Cardiology Unit, Interventional Cardiology Department, Hospital de la Santa Creu I Sant Pau, 08025 Barcelona, Spainlasmarats@santpau.cat (L.A.);; 2 School of Medicine, Universitat Autónoma of Barcelona, 08193 Barcelona, Spain; 3 Interventional Cardiology Unit, Hospital Alvaro Cunqueiro, 36312 Vigo, Spain; 4 Heart Area, Hospital Universitario Central de Asturias, 33011 Oviedo, Spain; 5 Cardiovascular Institute, Hospital Clinico San Carlos, IdISSC, 28040 Madrid, Spain; 6 Department of Cardiology, University Hospital of Leon, 24008 Leon, Spaincgarrote@saludcastillayleon.es (C.G.);; 7 Department of Cardiology, Parc Taulí Hospital Universitari, 08208 Sabadell, Spain

**Keywords:** Mitral regurgitation, left ventricular global longitudinal strain, MitraClip

## Abstract

Background: The timing and selection of optimal candidates for mitral transcatheter edge-to-edge valve repair remains to be fully determined, especially in cases with severely depressed left ventricular ejection fraction (LVEF). The objective of this study is to evaluate the prognostic value of myocardial strain (LVGLS) in this setting. Methods: Retrospectively, 172 consecutive patients with LVEF ≤40% and severe MR treated with MitraClip were included. Four groups were generated according to the LVEF (<30% or **≥**30%) and median LVGLS. The primary end-point was cardiovascular mortality. Results: Procedural success was high (96.5%) and complications were rare. At one-year follow-up, 82.5% of patients maintained MR grade ≤2, 79.2% were at a NYHA class ≤II and a reduction of 80% in heart failure admissions was observed in all groups. Interestingly, among patients with a more depressed LVEF, LVGLS was found to be an independent predictor for cardiovascular mortality (HR: 3.3; 95% CI: 1.1–10, *p* = 0.023). Conclusions: Mitral valve repair with MitraClip is safe and it improves the mid-term functional class of patients regardless of LVEF. LVGLS can help in the selection of optimal candidates and timing for this procedure, as well as in the recognition of those patients with worse prognoses.

## 1. Introduction

Mitral valve regurgitation (MR) is nowadays the second most common valvular heart disease and is associated with a deterioration of the quality and expectancy of life of those who suffer from this condition. The European Society of Cardiology recommends surgical treatment if severe and symptomatic MR is present or if there are signs of advanced impact of the disease such as the development of atrial fibrillation, pulmonary artery hypertension or enlargement of the left ventricle or atrium [1]. However, a non-neglectable percentage of these patients are deemed to be at high surgical risk due to comorbidities or reduced left ventricular ejection fraction (LVEF) [2]. 

The MitraClip System (Abbott Vascular, Abbot Park, Illinois), a transcatheter technique that mimics the Alfieri stitch [3] has arisen as an alternative to surgery for high-risk patients and has demonstrated its safety and efficacy in reducing MR severity and improving symptoms and quality of life [4,5,6,7]. In recent years, there has been a growing interest in defining the optimal candidates and timing for this therapy, particularly since the controversial results from MITRAFR (Multicenter Study of Percutaneous Mitral Valve Repair MitraClip Device in Patients With Severe Secondary Mitral Regurgitation) and COAPT (Cardiovascular Outcomes Assessment of the MitraClip Percutaneous Therapy for Heart Failure Patients With Functional Mitral Regurgitation) trials were reported [8,9,10]. It has been hypothesized that there could be a “point of no return” at which this therapy may lack clinical benefit despite being technically feasible [11]. This may be of special interest in the presence of reduced LVEF where the possibility of determining the potential ability of the myocardium to adapt to new loading conditions after MitraClip implantation may identify good candidates for the procedure. In this regard, assessing the myocardial deformation through the quantification of the strain, a parameter that is less affected by preload and afterload and more reproducible [12,13] could add incremental value to the decision-making process [14,15,16,17,18]. 

This study sought to evaluate the potential predictor capacity of myocardial strain in patients affected with severe MR and reduced LVEF undergoing mitral transcatheter edge-to-edge repair (M-TEER) with the MitraClip System. 

## 2. Materials and Methods

Data was obtained from the Spanish MitraClip multicenter registry which included all consecutive patients affected with symptomatic MR grade ≥3 undergoing M-TEER since June 2012 in 24 participating centers. This study evaluated the data from all patients with baseline LVEF ≤ 40% enrolled in five of these institutions. All patients were deemed to be at high risk for surgery and considered for percutaneous repair after evaluation by the local Heart Team. The study was performed in accordance with the local ethics committee and all patients signed informed consent for the procedure. 

Baseline demographic parameters included age, gender, weight, height, hypertension, diabetes mellitus, renal impairment, chronic hemodialysis, smoking, atrial fibrillation, previous cerebrovascular disease, ischemic heart disease, New York Heart Association (NYHA) functional class, Society of Thoracic Surgeons (STS) score, and heart failure hospitalization during the year prior to the procedure. Patients were considered to be under optimal medical treatment if in compliance with the guidelines valid at the time of the procedure.

Baseline echocardiographic parameters were based on pre-procedural transthoracic (TTE) and transesophageal (TEE) echocardiography following the European Association of Cardiovascular Imaging recommendations [19,20] and performed by the local expert on cardiovascular imaging. Left ventricular ejection fraction (LVEF) was assessed by echocardiography using the biplane Simpson method. The severity of pre-procedural MR was assessed taking into account color flow Doppler, vena contracta width, the flow convergence method, pulmonary venous flow and continuous wave doppler. Post-procedural or residual MR was assessed taking into account the number of jets, color flow doppler, vena contracta width, continuous wave doppler and pulmonary venous flow [21]. Strain analysis was performed offline using 2D strain imaging software QLAB Advanced Quantification Software 13.0, Koninklijke Philips Electronics NV 2019 and the General Electric Automated Function Imaging software. The software automatically traced the endocardial borders at the end of systole with further manual adjustments if necessary to optimized automated speckle tracking. 

Technical success was defined as the correct implantation of at least one device and the absence of procedural mortality or emergent cardiovascular intervention related to the procedure while procedural success was defined as the achievement of MR grade reduction to 2 or less [22]. Procedure-related bleeding and its severity were defined according to the BARC (Bleeding Academic Research Consortium) criteria [22]. 

NYHA class, MR grade, mean mitral gradient, pulmonary artery systolic pressure (SPAP), LVEF, left and right ventricular dimensions, left atrial volume and left ventricular global longitudinal strain (LVGLS) were recorded as well as the need for subsequent mitral valve surgery, heart transplant, heart failure rehospitalization and mortality. Major composite clinical events (MACE) were a composite of cardiovascular death and rehospitalization due to heart failure at follow-up.

The assessment of normality was performed using the Shapiro–Wilk test. Results are presented as the mean (standard deviation) for continuous variables with a normal distribution, the median (interquartile range) for continuous variables with a non-Gaussian distribution, and with counts and percentages in the case of categorical variables. We divided our cohort into four groups according to the degree of LV dysfunction (severe dysfunction if LVEF was below 30% or moderate dysfunction if LVEF was above or equal to 30%) and LVGLS median value (−8.4%). Group 1 was comprised of patients with an LVEF above 30% and an LVGLS < −8.4%; group 2 included patients with LVEF < 30% and LVGLS < −8.4%; group 3 restrained patients with LVEF **≥** 30% and LVGLS **≥ −8.4%**; and group 4 incorporated patients with LVEF **<** 30% and LVGLS **≥ −8.4%**. Differences between groups were tested using the chi-square test for categorical variables and Student’s *t* and ANOVA tests for continuous variables. A 95% level of significance was applied (*p* < 0.05). Kaplan–Meier survival estimator was used to determine the time to MACE at follow-up. Data from survival curves and event-free survival rates along with the long-rank test were used for comparison between groups. Multivariate analysis testing the association of the different parameters studied was performed using Cox proportional hazard modeling. For data analysis, the STATA 13.1 version statistical package was used (StataCorp LP, College Station, TX, USA).

## 3. Results

### 3.1. Baseline Results

A total of 275 patients with symptomatic MR and LVEF ≤ 40% were treated with the MitraClip system during the study period. Patients with unavailable baseline LVGLS were excluded (*n* = 103) resulting in a final study population of 172 patients. Baseline demographic and clinical characteristics are shown in Table 1. Most patients were male (73.4%), smokers (57.8%), and presented with hypertension (70.5%) as well as a history of ischemic cardiomyopathy (63%). The majority were at NYHA class III or IV at baseline (58.1% and 23.9%, respectively), and up to 82.1% had been hospitalized due to heart failure in the year prior to the intervention. There were no differences between groups in baseline clinical characteristics. 

Baseline echocardiographic parameters are summarized in Table 2. All patients had an LVEF ≤40% prior to the procedure (mean 32 ± 6%) and MR grade 3 or 4 (17.3% and 82.7% respectively). Median LVGLS was −8.4% (IQ: −6.2% to −11.3%). The mean end-diastolic left ventricle diameter (EDLVD) was 64 ± 8 mm and the mean end-systolic left ventricle diameter (ESLVD), 48 ± 11 mm. As expected, patients in groups 2 and 4 presented larger left ventricular end-diastolic and end-systolic volumes and diameters than those from groups 1 and 3. Most MR was purely functional (71.7%) while in 17.3 % the mechanism of the MR was deemed to be mixed with non-significant differences between groups. Other echocardiographic characteristics of the mitral valve status such as the effective regurgitant orifice area (EROA), the presence of calcification, posterior leaflet retraction, or the location and number of jets were evenly distributed among groups. 

Right ventricular function assessed by TAPSE (tricuspid annular plane systolic excursion) was slightly more impaired in groups 3 and 4 in comparison with groups 1 and 2 (17 ± 4 mm and 16 ± 4 mm vs. 18 ±4 mm, respectively, *p* = 0.011) even though there were no differences in the fractional area change. Right ventricular, diameter was larger in groups 1 and 2 (50 ± 13 and 51 ± 15 mm) than in patients of groups 3 and 4 (42 ± 8 and 46 ± 17 mm, *p* = 0.040). 

### 3.2. Procedural Results

The procedure was highly successful in all subgroups with an overall procedural success rate of 96.5%. Two-thirds of the clips implanted were first and second generation and 33% of the clips implanted were third-generation. Procedural complications were infrequent and mostly related to vascular access bleeding (Table 3). One patient died in the first 24 h due to heart failure and two patients presented with a stroke during the first 24 h post-procedure.

### 3.3. Follow-Up Results

Median follow-up was 711 days (IQR:280–1250 days). 

**Figure 1:**
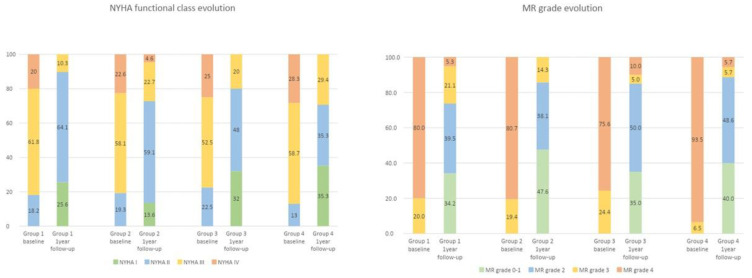
NYHA functional class and MR grade evolution (data from baseline and at one-year follow-up) in the different groups.

At one-year follow-up, 82.5% of patients remained with MR grade ≤2 and 79.2% in NYHA functional class ≤II (Figure 1). Although the percentage of patients with a maintained MR grade ≤2 was numerically lower in group 1 than in the rest of the groups, this difference did not reach statistical significance (*p* = 0.425). Twelve-month NYHA class was higher in groups 2 and 4, although it did not reach statistical significance (*p* = 0.135). Throughout the study period, 14% of the patients were admitted to the hospital due to heart failure, translating into a reduction of nearly 80% in re-hospitalizations as compared to the rate of hospitalizations the year before the procedure. 

**Figure 2:**
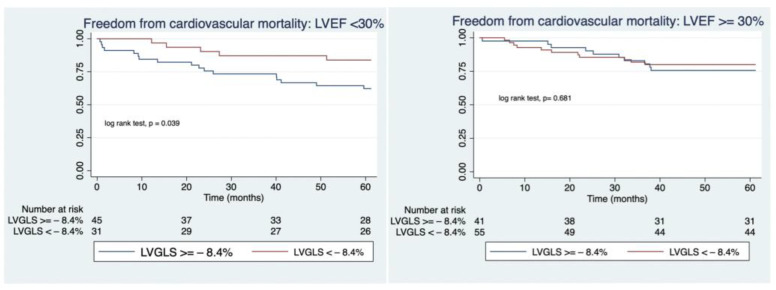
Kaplan–Meier freedom from cardiovascular mortality.

**Figure 3:**
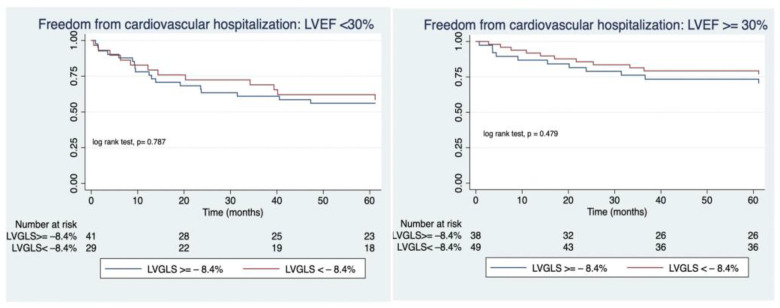
Kaplan-meier freedom from re-hospitalization due to heart failure.

Thirteen patients (7.5%) died during this period due to cardiovascular reasons and two patients (1.18%) received a cardiac transplant. Adjusted Cox model regression survival analysis yielded that a worse value of LVGLS was an independent predictor of cardiovascular mortality in patients with a more altered LVEF (HR: 3.3; 95% CI: 1.1–10, *p* = 0.023) while in those with a more preserved LVEF, LVGLS was not a predictor of cardiovascular mortality (OR:1.1; 95% CI:0.3–3.1; *p* = 0.956), (Table 4). Kaplan–Meier analysis curves illustrate this finding, as shown in Figure 2. However, when evaluating the rate of re-admission due to heart failure, there were no differences regarding the status of LVGLS in either the patients with a higher LVEF nor those with alower LVEF (Figure 3).

## 4. Discussion

The main findings of this study are: (1) M-TEER with the MitraClip system is safe even in the presence of left ventricle dysfunction; (2) the procedure is effective and its mid-term durability is high; and (3) left ventricular global longitudinal strain provides valuable prognostic information, especially in patients with a more impaired left ventricular ejection fraction.

Nowadays, MitraClip has proven to be a relatively safe therapy with high procedural effectiveness when applied to selected patients in high-volume centers [6,7,9,10]. Our study shows that this affirmation also applies to selected patients with poor left ventricular performance—measured as those with LVEF ≤ 40%—in whom the procedure was found to be successful in 97% of cases and in whom we found out a very low peri-procedural mortality rate (0.6%) as well as a low complication rate that was mainly driven by vascular access bleeding (4.6%). However, it should be noted that all patients had been previously evaluated by the local heart team ensuring not only a technical plausibility of the repair but also pre-procedural guideline-directed medical therapy. 

Nevertheless, despite its safety, the main concern about this technique in this setting is its mid-term durability and whether the improvement in MR grade is translated into an improvement in quality and expectancy of life. While several reports have pointed out that M-TEER could be safe and effective regardless of LVEF [23] others have published different results that may indicate that the treatment may be futile if advanced left ventricular dysfunction is already present [9,24,25,26,27]. These controversial results have directed the focus of attention toward the pre-selection of patients and currently, efforts are being made in order to define the optimal candidates for this therapy. In this way, the analysis of myocardial deformation with the evaluation of global longitudinal strain of the left ventricle (LVGLS) could be of interest as it has already been proven that this tool enables more precise examination of the cardiac muscle performance. Furthermore, LVGLS could also provide with valuable information as it has been found to be a good predictor of mortality in patients with reduced LVEF [18] as well as of left ventricular remodeling six months after repair with MitraClip [27,28].

In our cohort, the reduction in MR grade was highly sustained over time as more than 80% of the patients maintained a mild regurgitation (MR grade I or II) at one-year of follow-up. More importantly, at that time nearly 80% of the patients were at NYHA class I or II which is a substantial improvement in quality of life since more than 82% of patients were at NYHA class III or IV prior to the procedure, being that result in line with previous studies [5,6,10]. Of note, the percentage of rehospitalizations due to cardiac failure within the first year of follow-up decreased significantly in comparison with the rate of heart failure-related admissions the year prior to the repair (14% vs. 82%, respectively). This reduction was observed both in the group with a more diminished LVEF as well as in the group with a more preserved LVEF regardless of baseline LVGLS as shown in Figure 3. However, when comparing the cardiac mortality at one-year follow-up, we found out that while those patients with a lower LVEF and a more deteriorated LVGLS had a significantly higher rate of cardiac mortality as compared with those with a less impaired LVGLS, in the subgroup of patients with a baseline LVEF **≥** 30%, the status of LVGLS was not a predictor of events (Figure 2). It could be argued that LVGLS could be more altered in patients with larger left ventricular diameters and volumes, which already have a poorer prognosis, and therefore, its additional value, could be questionable. In order to examine this concern, we studied the correlations between LVGLS and end-diastolic left ventricular diameter (EDLVD) and end-diastolic left ventricular volume (EDLVV). In our population, LVGLS was not correlated neither with EDLVD or EDLVV (rho = −0.007, *p* = 0.933 and rho= −0.016, *p*= 0.33 respectively) which confers the assessment of LVGLS an additional value in terms of prognosis. These findings translate that LVGLS could be an indicator of more advanced deterioration of the cardiac muscle that, even if an amelioration of the volume overload occurs, may not be able to adapt to the new loading condition, especially in severely depressed LVEF cases. Therefore, proper patient assessment, including the evaluation of myocardial deformation using LVGLS, could lead to the recognition of patients who may benefit the most and it could also lead to prompt therapy in those patients with progressive worsening of left ventricular function in terms of progressive worsening of LVGLS. However, larger and randomized trials are needed to fully examine the benefits of this approach. 

There are several limitations to be considered in this paper. The first one is its own nature as an observational and retrospective study with a lack of a control group to compare outcomes. 

Second, during the period of study, the European Society of Cardiology’s recommendations in terms of oral treatment for heart failure changed according to new data available and thus, patients included at the beginning of the study were under different medication than the ones included later [29,30,31].

Third, the lack of core-lab adjudication of echocardiographic parameters could be considered a limitation. However, it reflects a real-world situation in which a local expert in cardiac imaging performs the acquisition and evaluation of the baseline echocardiograms as well as monitors the procedure and post-procedural echocardiograms. Moreover, the software used to calculate LVGLS was automatic requiring minimal adjustments to optimized automated speckle tracking, thus, reducing interobserver and interinstitutional variability. Despite this, in order to assess interobserver variability, 17 patients were randomly selected and the LVGLS was assessed by two different observers obtaining a kappa index of 0.946, *p* < 0.001. 

Fourth, the availability of the required images for the analysis of the baseline myocardial strain is not high in our cohort (63% of the initial population) and it could have caused a selection bias as a pure secondary cause of MR was more frequently found in patients without LVGLS availability than in those with LVGLS availability (Supplemental Tables S1 and S2).

Last, two different softwares for the analysis of LVGLS were used as per availability in the participating centers. Since there has been reported an inter-vendor variability in the values of LVGLS, care should be taken in interpreting a specific cut-off point or value. 

## 5. Conclusions

Transcatheter edge-to-edge mitral repair with the MitraClip system is safe and it improves the mid-term quality of life of patients regardless of LVEF. Myocardial deformation evaluated by means of LVGLS can help in the selection of optimal candidates and timing for this procedure, as well as to recognize those patients with worse prognoses.

## Figures and Tables

**Table 1:** Basal demographic and clinical characteristics.

	Overall	Group 1 *n* = 55	Group 2 *n* = 31	Group 3 *n* = 41	Group 4 *n* = 46	*p*
Age (years)	72.5 ± 9.6	75.5 ±9.6	70.3 ± 8.2	71.7 ± 11.4	72.4 ± 8.3	0.133
Sex (Male %)	73.4	70.9	64.5	80.5	76.1	0.448
Hypertension (%)	70.5	70.9	61.3	73.2	73.9	0.644
Diabetes Mellitus (%)	46.8	45.5	58.0	41.5	45.7	0.549
Glomerular filtrate rate <60 (%)	47.7	49.1	63.3	41.5	41.3	0.224
Smoking (%)	57.8	56.4	70.9	63.4	45.7	0.135
Hemodialysis (%)	0.6	0.0	3.2	0.0	0.0	0.203
Atrial Fibrillation (%)	54.3	65.5	45.2	46.3	54.4	0.183
Previous stroke (%)	14.5	18.2	6.5	14.6	15.2	0.523
Ischemic cardiomyopathy (%)	63	61.8	54.8	75.6	58.7	0.237
Previous PCI (%)	41.3	29.1	43.3	53.7	43.5	0.107
Previous CABG (%)	17.3	14.6	9.7	19.5	23.9	0.378
Previous valvular intervention (%)	10.4	5.5	6.5	14.6	15.2	0.273
NYHA Class (%)						0.899
1	0	0.0	0.0	0.0	0.0	
2	18	18.2	19.3	22.5	13.0	
3	58.1	61.8	58.1	52.5	58.7	
4	23.9	20.0	22.6	25.0	28.3	
Previous Year Heart Failure Admission (%)	82.1	80.0	80.7	82.9	84.8	0.929
ProBNP	5278 (2810–7990)	4803.5 (1892–8648)	5509 (5017–6002)	6012 (3000–9210)	2899 (2515–6104)	0.777
STS score	4.6 ± 3.5	4.6 ± 3.1	3.3 ± 2.4	4.6 ± 3.7	5.0 ± 4.2	0.406
EuroScore II	8.3 ± 5.5	7.2 ± 5.8	7.0 ± 5.0	8.2 ± 7.7	9.8 ± 5.5	0.377

PCI: percutaneous cardiac intervention; CABG: coronary artery bypass grafting; NYHA: New York Heart Association; ProBNP: pro-brain natriuretic peptide; STS: Society of Thoracic Surgeons.

**Table 2:** Basal echocardiographic characteristics.

	Overall	Group 1 *n* = 55	Group 2 *n* = 31	Group 3 *n* = 41	Group 4 *n* = 46	*p*
LVEF (%)	32 ± 6	37 ± 3	25 ± 4	36 ± 2	25 ± 5	<0.000
EDLVV (mL)	193 ± 65	163 ± 47	278 ± 64	183 ± 59	203 ± 67	0.018
ESLVV (mL)	127 ± 53	95 ± 35	194 ± 56	117 ± 42	147 ± 55	0.004
EDLVD (mm)	64 ± 8	61 ± 8	67 ± 8	61 ± 7	67 ± 9	<0.000
ESLVD (mm)	48 ± 11	43 ± 9	58 ± 6	47 ± 10	55 ± 10	0.000
MR Grade (%)						0.096
≤2	0	0.0	0.0	0.0	0.0	
3	17.3	20.0	19.4	24.4	6.5	
4	82.7	80.0	80.7	75.6	93.5	
MR mechanism (%)						0.075
Primary	4	9.1	0.0	4.9	0.0	
Secondary	71.7	63.5	64.5	70.7	84.8	
Mixed	24.3	25.5	35.5	24.4	15.2	
Mitral annulus calcification (%)	26.6	32.7	19.4	32.5	17.4	0.401
Subvalvular apparatus calcification (%)	8.2	5.7	3.7	5.6	16.7	0.139
Posterior leaflet retraction (%)		61.8	61.3	57.5	56.5	0.941
Excentric jet (%)	17.4	27.3	19.4	7.5	13.0	0.068
Multiple jets (%)	10.7	14.6	3.5	0.0	19.6	0.012
EROA (cm)	0.4 ± 0.2	0.4 ± 0.2	0.4 ± 0.1	0.5 ± 0.2	0.4 ± 0.2	0.187
Mitral Mean Gradient (mmHg)	1.5 ± 0.6	1.7 ± 0.8	1.5 ± 0.7	1.5 ± 0.6	1.3 ± 0.4	0.474
Mitral Valvular Area (cm^2^)	5.7 ± 1.4	5.8 ± 1.3	5.4 ± 1.6	5.6 ± 1.3	5.9 ± 1.9	0.874
Systolic Pulmonary Artery Pressure (mmHg)	48 ± 15	48 ± 13	44 ± 15	51 ± 17	46 ± 14	0.250
LVGLS (%)	8.4 (6.2–11.3)	11.3 (10–12.8)	11.4 (9.6–12.3)	5.7 (5–6.8)	6.9 (5–7.7)	<0.000
Tricuspid regurgitation grade (%)						0.197
0–1	52	64.0	40.0	50.0	50.0	
2	15.7	16.0	20.0	21.4	8.8	
3	19.6	4.0	33.3	17.9	26.5	
4	12.8	16.0	6.7	10.7	14.7	
TAPSE (mm)	17 ± 4	18 ± 4	18 ± 4	17 ± 4	16 ± 4	0.190
Right Ventricle Fractional Area Change (%)	40 ± 12	38 ± 11	40 ± 15	43 ± 10	40 ± 16	0.570
EDRVD (mm)	47 ± 13	50 ± 13	51 ± 15	42 ± 8	46 ± 17	0.040
LAV (mL)	106 ± 55	118 ± 41	104 ± 39	104 ± 43	96 ± 29	0.258

LVEF: left ventricular ejection fraction; EDLVV: end-diastolic left ventricular volume; ESLVV: end-systolic left ventricular volume; EDLVD: end-diastolic left ventricular diameter; ESLVD: end-systolic left ventricular volume; EROA: effective regurgitant orifice area; LVGLS: left ventricular global longitudinal strain; EDRVD: End-diastolic right ventricular diameter; LAV: left atrium volume.

**Table 3:** Procedural outcomes.

	Overall	Group 1 *n* = 55	Group 2 *n* = 31	Group 3 *n* = 41	Group 4 *n* = 46	*p*
Technical success (%)	99.4	98.2	100.0	100.0	100.0	0.540
Procedural success (%)	96.5	96.4	100.0	100.0	91.3	0.096
Number of Clips placed						0.834
1	64.2	67.3	67.7	58.5	63.0	
2	28.3	29.1	25.8	31.7	26.1	
3	7.5	3.6	6.5	9.8	10.9	
≥4	0	0.0	0.0	0.0	0.0	
Partial/Complete Detachment (%)	1.2	0.0	6.4	0.0	0.0	0.073
Embolization (%)	0	0.0	0.0	0.0	0.0	N/A
Chords Rupture (%)	0.6	1.8	0.0	0.0	0.0	0.544
Conversion to surgery (%)	0.1	0.0	0.0	0.0	0.0	
Major bleeding MVARC scale (%)	4.6	1.8	6.4	9.8	2.2	0.232
Cardiac Tamponade (%)	0	0.0	0.0	0.0	0.0	N/A
Residual MR (%)						0.282
0	2.3	1.8	0.0	7.3	0.0	
1	50.3	54.6	51.6	48.8	45.7	
2	42.8	36.4	48.4	43.9	45.7	
3	2.9	3.6	0.0	0.0	6.5	
4	1.7	3.6	0.0	0.0	2.2	
Residual Mitral Mean Gradient (mmHg)	2.8 (2–4)					
Stroke (%)	1.2	1.8	0.0	2.4	0.0	0.636
Peri-operative mortality (<24 h) (%)	0.6	0.0	0.0	0.0	2.2	0.427

**Table 4:** Adjusted Cox regression analysis for cardiovascular mortality.

	LVEF ≥ 30%		
	HR	95% CI	*p* Value
Ischemic Cardiomyopathy	2.0	0.7–6.2	0.211
Glomerular filtrate rate < 60	2.3	0.9–5.9	0.880
Previous Year Heart Failure Admission (%)	0.7	0.2–1.9	0.442
LVGLS	1.1	0.3–3.1	0.956
SPAP	0.4	0.1–1.1	0.060
EDRVD	1.1	0.3–3.2	0.941
	LVEF < 30%		
	HR	95% CI	*p* value
Ischemic Cardiomyopathy	1.7	0.6–4.4	0.288
Glomerular filtrate rate < 60	1.4	0.5–3.6	0.502
Previous Year Heart Failure Admission (%)	1.4	0.4–5.3	0.599
LVGLS	3.3	1.4–10	0.023
SPAP	0.9	0.4–2.4	0.856
EDRVD	2.8	0.8–10.6	0.134

LVGLS: Left ventricular global longitudinal strain; SPAP: systolic pulmonary artery pressure; EDRVD: End diastolic right ventricular diameter.

## Data Availability

The data presented in this study are available on request from the corresponding author.

## Supplementary Materials

The following supporting information can be downloaded at: https://www.mdpi.com/article/10.3390/jcm12124116/s1, Table S1: Baseline clinical characteristics in patients with LVGLS available and those with no LVGLS available; Table S2. Baseline clinical characteristics in patients with LVGLS available and those with no LVGLS available; Figure S1. Example of analysis of left global longitudinal strain being measured.
