# Supplementary material for: Global Longitudinal Strain Predicts Outcomes in Patients with Reduced Left Ventricular Function Undergoing Transcatheter Edge-to-Edge Mitral Repair
Source: J Clin Med. 2023 Jun 18;12(12):4116. doi: 10.3390/jcm12124116 (PMC10298909; doi:10.3390/jcm12124116)

SUPPLEMENTAL MATERIAL

Title: Global longitudinal strain predicts outcomes in patients with reduced left ventricular function undergoing transcatheter edge-to-edge mitral repair

Table S1

	**Overall**	**LVGLS available n=172**	**LVGLS non available n=103**	**p**
**Age (years)**	73.1 (67.5-79.8)	73 (69-79)	73.2 (67-79.9)	0.690
**Sex (Male %)**	71.3	73.4	67.7	0.310
**Hypertension (%)**	73.1	70.5	77.5	0.211
**Diabetes Mellitus (%)**	48.4	47	51	0.510
**Glomerular filtrate rate <60 (%)**	49.1	47.4	51.5	0.543
**Smoking (%)**	60.7	57.8	65.7	0.194
**Hemodialysis (%)**	1.1	0.6	2	0.297
**Atrial Fibrillation (%)**	54	54.3	54	0.947
**Previous stroke (%)**	12.7	14.5	9.8	0.256
**Ischemic cardiomyopathy (%)**	64.4	63	66.7	0.539
**Previous PCI (%)**	44.5	41.3	50	0.160
**Previous CABG (%)**	18.2	17.3	19.6	0.639
**Previous valvular intervention (%)**	9.1	10.4	6.9	0.315
**NYHA Class (%)**				0.883
**I**	0	0	0	
**II**	17.1	18	15.7	
**III**	58.8	58.1	59.8	
**IV**	24.1	23.8	24.5	
**Previous Year Heart Failure Admission (%)**	78.6	82.1	72.6	0.070
**ProBNP**	5017 (2906-9210)	5279 (2810-7990)	5000 (3931-11150)	0.308
**STS score**	3.6 (1.9-6.2)	3.6 (1.8-6.2)	3.7 (2.1-6.3)	0.375
**EuroScore II**	5.4 (2.9-9.3)	5.4 (2.8-9)	5.4 (3-9.8)	0.855

PCI: percutaneous cardiac intervention; CABG: coronary artery bypass grafting; NYHA: New York Heart Association; ProBNP: pro-brain natriuretic peptide; STS: Society of Thoracic Surgeons.

Table S2

	**Overall**	**LVGLS available n=172**	**LVGLS non available n=103**	**p**
**LVEF (%)**	31.6 (26.3-36.0)	33 (27-36.2)	30 (26-36)	0.088
**EDLVV (ml)**	180 (140-224)	186 (143-237)	161 (131-207)	0.162
**ESLVV (ml)**	110 (85-155)	118 (90-158)	104 (78-155)	0.162
**EDLVD (mm)**	62 (58-69)	63 (58-69)	62 (56-69)	0.493
**ESLVD (mm)**	48 (42-57)	45 (39-57)	48 (44.5-57)	0.152
**MR Grade (%)**				0.586
**≤2**	0	0	0	
**3**	18.3	17.3	20	
**4**	81.7	82.7	80	
**MR mechanism (%)**				**<0.000**
**Primary**	4.4	4.1	4.9	
**Secondary**	61.5	24.3	51	
**Mixed**	34.2	71.7	44.1	
**Mitral annulus calcification (%)**	26.6	26.2	28.4	0.816
**Subvalvular apparatus calcification (%)**	7	8.2	5.1	0.331
**Excentric jet (%)**	20.5	17.4	25.7	0.105
**Multiple jets (%)**	8.9	10.7	6	0.184
**EROA (cm)**	0.4 (0.3-0.6)	0.4 (0.3-0.5)	0.5 (0.3-0.6)	0.060
**Mitral Mean Gradient (mmHg)**	1.5 (1-2)	1.3 (1-2)	1.9 (1-2.1)	0.060
**Mitral Valvular Area (cm2)**	5.5 (4.5-6.7)	5.5 (4.5-6.7)	5.5. (4.5-6.7)	0.859
**Sistolyc Pulmonary Artery Pressure (mmHg)**	48 (35-58)	50 (39-57)	45 (34-60)	0.667
**LVGLS (%)**	8.8+/-2.6			N/A
**Tricuspid regurgitation grade (%)**				0.091
**0-1**	51.9	52	51.7	
**2**	18.8	15.7	29	

**3**	17.3	19.6	9.7	
**4**	12	12.8	9.7	
**TAPSE (mm)**	17±4	17±4	16.9±4	0.839
**Right Ventricle Fractional Area Change (%)**	40±13.8	40.2±12.5	39.9±17.9	0.954
**EDRVD (mm)**	46.3±12.6	47±13.4	44.2±11	0.123
**LAV (ml)**	103.9±52.7	106.7±55.4	95.1±45.9	0.155

LVEF: left ventricular ejection fraction; EDLVV: end-diastolic left ventricular volume; ESLVV: end-systolic left ventricular volume; EDLVD: end-diastolic left ventricular diameter; ESLVD: end-systolic left ventricular volume; EROA: effective regurgitant orifice area; LVGLS: left ventricular global longitudinal strain; EDRVD: End-diastolic right ventricular diameter; LAV: left atrium volume.

Figure S1. Example of analysis of left ventricular global longitudinal strain being measured

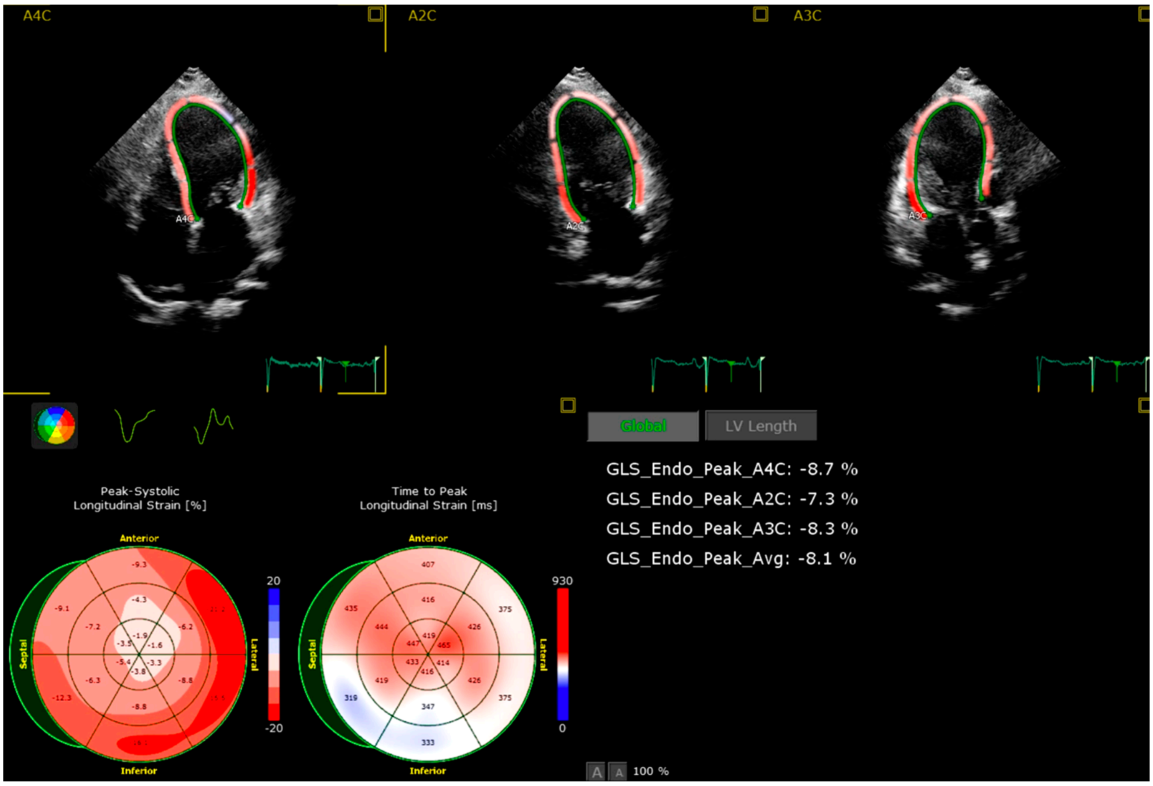

Supplement: Supplementary file 1 [file jcm-12-04116-s001.zip › jcm-2439194-supplementary.pdf]
